# Event and Comment

**Published:** 1912-05-15

**Authors:** 


### DENTAL REGISTER.

**Vol. LXVI. May 15, 1912. No. 5**

### EVENT AND COMMENT.

EXCESSES IN EATING. We are reprinting an article containing much good advice on the question of properly selecting one's food and not abusing one's powers of assimilation by over indulgence. The article is the result of a long study and personal experience of Dr. S. M. Stauffer of Pittsburgh, who has the distinction of controlling his desires for eating and almost breaking the fasting record. There is much in the article that should claim our attention, and probably some of the statements would have to be restated by other experiments, as "one man's food may be another's poison." Of course it is not the dentist's sphere to advise people what to eat, except perhaps as to feeding during the formative periods of infancy when the teeth and jaws are being built, but for his own benefit, he should know how to keep himself in proper condition to perform his daily duties. There is one paragraph we shall quote as it has an important bearing on the good health of the dentist particularly and needs emphasizing.

"If we were all leading a normal life—spending plenty of time in the open air, Fletcherizing our foods, not using all our stored up energy in the "strenuous life," having peace of mind and perfect poise, harmonious surroundings, pleasurable forms of exercise in pure air with proper methods of breathing, and eating only when physiological hunger asserts itself, and to the extent of satisfying hunger and not appetite, —then only would we be likely to select our food with intelligence and mal-nutrition would need little attention."

The qualifying clause in the first sentence of this quotation saves us from feeling the full force of the statement, very few persons who are doing a full days work in these strenuous times could comply with the catalog of conditions named. It is hoped that many can boast some of them. We recently saw a newspaper comment on the tremendous work of a prominent man who had accomplished a large work and died when he was only fifty-years of age. There was a note to the effect that he had been raised on a farm and had a splendid physicial constitution to sustain him in his great work. We could not refrain from the comment that had he spent some of his great talent on his physical preservation he could have lived longer and accomplished even more than he did in his short life. He lived to achieve, and to do this he had to concentrate his whole being into the objective before him. . But he had to eat to have at his disposal the energy demanded for such high pressure service, and he had to eat probably more than he used, because he had no time to weigh out in exact caloric values the several kinds of food he needed for each day. He did not know how, and neither did he know how much energy he should need for each days work, as he never worked by the *clock* or the *piece.* So he found it safe to eat at each meal all that he wanted of what was placed before him,—usually much more than he would be able to assimilate, consequently he not only tired out his muscles and nerves by over work, but also his. digestive functions, and worst of all his organs of eliminating the waste products and the ingested surplus gradually weakened and fermentation, putrifaction and intoxication followed and the end was death.

Is there any lesson here for the busy dentist who stops for an hour in the middle of the day to eat a hearty dinner, which his afternoon occupation gives him no chance to digest or assimilate? We must have all the food we need and a little more for waste perhaps, but most of us eat too much.

